# Enhanced Anti-melanoma Efficacy of a Pim-3-Targeting Bifunctional Small Hairpin RNA via Single-Stranded RNA-Mediated Activation of Plasmacytoid Dendritic Cells

**DOI:** 10.3389/fimmu.2019.02721

**Published:** 2019-11-26

**Authors:** Jing Liu, Yuan Hu, Qie Guo, Xin Yu, Liwei Shao, Cai Zhang

**Affiliations:** Institute of Immunopharmaceutical Sciences, School of Pharmaceutical Sciences, Shandong University, Jinan, China

**Keywords:** melanoma, Pim-3, single-stranded (ssRNA), plasmacytoid dendritic cells, tumor immunotherapy

## Abstract

Melanoma is the most serious type of skin cancer. The immunosuppressive tumor microenvironment and aberrant expression of some proto-oncogenes are the main cause of melanoma development. We have constructed a single-stranded RNA (ssRNA)–Pim-3–small hairpin RNA (shRNA) dual-function vector, which activates the toll-like receptor (TLR)7 to stimulate the antitumor immune response through ssRNA fragments and simultaneously silences the proto-oncogene Pim-3 to intensify apoptosis of the tumor cells via shRNA. Here, we found that therapy with the ssRNA-Pim-3-shRNA dual-function vector not only promotes the apoptosis and inhibits the proliferation of B16F10 melanoma cells by inhibiting the expression of Pim-3 but also enhances the activation of CD8^+^ T cells and natural killer (NK) cells and simultaneously reduces the proportion of intratumoral regulatory T cells (Tregs) and myeloid-derived suppressor cells (MDSCs). Together, these features effectively inhibit the growth of melanoma. Intriguingly, the bifunctional therapeutic effect that reverses the tumor immunosuppressive microenvironment is dependent on the activation of plasmacytoid dendritic cells (pDCs) and the secretion of type I interferon (IFN). Our study suggests that ssRNA-Pim-3-shRNA dual-function therapy is expected to become a promising therapeutic strategy for melanoma and other solid tumors with immunosuppressive microenvironment.

## Introduction

Melanoma is the most serious form of skin cancer owing to the high degree of malignancy. Proto-oncogenes play a key role in the growth of melanoma (1). The abnormally high expression of the proto-oncogene causes changes in the biological characteristics of the cells, resulting in increased cell proliferation and reduced cell apoptosis (2). The proto-oncogene Pim-3 is a highly conserved serine/threonine kinase and plays an important role in many physiology and pathology processes, including cell proliferation, survival, and apoptosis (3–5). Its abnormal expression in a variety of cancers, including melanoma, leads to decreased apoptosis and increased proliferation through phosphorylation and inactivation of the proapoptotic BH3-only protein Bad, thus promoting the occurrence and development of tumors (6, 7). Inhibition of Pim-3 kinase or silencing of Pim-3 inhibits tumor growth and enhances apoptosis of tumor cells (8). Therefore, Pim-3 kinase might be a candidate therapeutic target for cancer.

The immunosuppressive microenvironment of melanoma contributes to its development. The microenvironment includes tumor-infiltrating immunosuppressive cells, such as regulatory T cells (Tregs) and myeloid-derived suppressor cells (MDSCs), and their associated cytokines transforming growth factor β (TGF-β) and interleukin 10 (IL-10), which impair T cell migration, survival, proliferation, and effector functions, leading to T cell dysfunction or exhaustion (9, 10). Toll-like receptor (TLR)7 and TLR8 are important members of the TLR family and recognize pathogen-associated molecular patterns (PAMPs) such as single-stranded RNA (ssRNA) of viruses or structures similar to nucleosides (11). Activation of TLR7 or TLR8 can induce type I interferon (IFN) and inflammatory responses through activation of downstream IRF7 and NF-κB signaling pathways (12). The production of type I IFN can further initiate the activation of natural killer (NK) and T cells directly or with the help of activated antigen-presenting cells (APCs) and therefore enhance both antiviral and antitumor immune responses (11, 13). Intriguingly, stimulation of TLR7/8 signaling with TLR7/8 agonists can subvert tumor-induced immunosuppression in several types of solid cancers and has shown great promise in tumor therapy (14–16).

Dendritic cells (DCs) are professional APCs and can be classified into conventional DC (cDCs) and plasmacytoid DCs (pDCs). pDCs play a major role in antiviral immunity and elicit both innate and adaptive immune responses by secreting massive amounts of type I IFN upon recognition of viral DNA or RNA by TLRs. However, in the tumor microenvironment of many cancers including melanoma, pDCs were found in non-activated or immunotolerance state with low ability to produce type I IFN (17, 18). Upon therapeutic activation of pDCs by TLR agonists, pDCs appear to be reactivated and can induce local tumor regressions (19, 20). Previously, we reported that therapy with a dual-function small hairpin RNA (shRNA) vector containing a Pim-3-silencing shRNA (sh-Pim-3) and a TLR7-stimulating ssRNA markedly suppressed the growth of murine hepatoma through activating CD4^+^ T cells and NK cells (8). In the present study, we showed that this dual-function therapy not only can promote apoptosis and inhibit the proliferation of B16F10 melanoma cells by inhibiting the expression of Pim-3 but also can stimulate the activation of pDCs by ssRNA to secrete a large amount of type I IFN, thereby enhancing the activity of CD8^+^ T cells and NK cells in a B16F10 tumor-bearing mouse model.

## Methods and Materials

### Cell Culture and Animal Model

B16F10 cells were cultured in Roswell Park Memorial Institute (RPMI)-1640 medium supplemented with 10% fetal bovine serum (FBS) at 37°C in a 5% CO_2_ incubator. Pathogen-free C57BL/6 mice were purchased from HFK Bioscience Co., Ltd. (Beijing, China). Experiments were performed according to the guidelines approved by the Committee on the Ethics of Animal Experiments of Shandong University. Injected subcutaneously were 5 × 10^5^ B16F10 cells into the right flank of C57BL/6 mice. The tumor mass was clearly identified at about 50 mm^3^ in size within 4 days. The mice were then intratumorally injected with dual-function, shRNA, and ssRNA vectors mixed with *in vivo*-jetPEI transfection reagent (Polyplus Transfection Inc., NY, USA) every 4 days, and the tumor volume was measured during 2 weeks of treatment. Tumor length and width were measured every 3 days, and the volume was calculated according to the formula (length × width^2^)/2.

### Plasmid Construction and Transfection

Plasmid construction of shRNA, ssRNA, and dual-function vectors was performed as described previously (8). The vectors were transfected into B16F10 cells using *in vitro*-jetPRIME transfection reagent (Polyplus Transfection Inc., NY, USA) according to the manufacturer's instructions.

### Measurement of Apoptosis

Annexin V-FITC/propidium iodide (PI) double staining kit (BestBio) was used to detect apoptosis of tumor cells via flow cytometry. Annexin V-positive cells represented the apoptotic cells. Alternatively, terminal deoxynucleotidyl transferase-mediated dUTP nick-end labeling (TUNEL) assay (C1089, Beyotime, Shanghai, China) was performed following the manufacturer's instructions. Briefly, the coverslips or tumor tissue sections were treated with proteinase K and incubated with TUNEL reaction mixture at 37°C for 1 h. Then the samples were incubated with 2-(4-amidinophenyl)-1*H*-indole-6-carboxamidine (DAPI) (C1002, Beyotime, Shanghai, China, 1/1000). The number of TUNEL-positive cells was calculated as percent of total number of cells.

### Flow Cytometry Analysis

To obtain tumor-infiltrating lymphocytes, the tumor tissues were peeled off and ground in a 200-mesh steel mesh. The supernatant was centrifuged at 700 rpm for 1 min to remove most of the tumor parenchymal cells. The supernatant was further centrifuged at 1,200 rpm for 5 min to obtain precipitated cells. The obtained cells were subjected to gradient density centrifugation with 40 and 70% Percoll solutions to obtain relatively pure tumor-infiltrating lymphocytes.

Tumor-infiltrating lymphocytes and splenic lymphocytes were isolated as described to analyze the percentages and activation of different lymphocyte populations. Briefly, after blocking non-specific Fc receptor (FcR) binding with anti-CD16/32 (eBioscience), the lymphocytes were stained with the indicated fluorescent mAbs for surface molecules. Then, cells were fixed, permeabilized, and stained with the mAbs against the indicated intracellular molecules or isotype control Abs. Table S1 lists the antibodies used. Cells were analyzed by fluorescence-activated cell sorting (FACS) Aria III (BD). Data were analyzed using the Flow Jo software (Treestar Inc., Ashland, OR, USA).

### Lymphocyte Depletion

mAbs purified from PK136 (α-NK1.1), 2.43 (α-CD8a), and GK1.5 (α-CD4) hybridoma cell lines were used to deplete NK, CD8^+^ T, and CD4^+^ T cells, respectively. One milligram of mAb was injected intraperitoneally into C57BL/6 mice for 7 days before inoculation with B16F10 cells. For depletion of pDC, mice were intraperitoneally injected with 250 μg of monoclonal antibody anti-CD317 (clone 927, Bio X cell, West Lebanon, USA) 1 week before inoculation with B16F10 cells. In order to ensure the efficiency of cell depletion, injection of the mAb was performed every 3 days during tumor treatment.

### Western Blotting

Western blotting was performed as described previously (8). The antibodies against Pim-3, Bcl-xl, Bcl-2, phosphorylated (p)-Bad, Bad, p-NF-κB, NF-κB, and β-actin were purchased from Cell Signaling Technology (Danvers, MA, USA).

### Quantitative Real-Time PCR

Total cellular RNA was extracted using a TRIzol RNA isolation kit (Invitrogen). Reverse transcription–PCRs were set up using M-MLV reverse transcriptase (TianGen). Samples were subjected to real-time PCRs on iCycler iQ Real-Time PCR System (Bio-Rad) using a SYBR Green kit (Roche). β-Actin was used as internal control. The sequences of the PCR primers are as follows: β-actin: 5′-AGAGGGAAATCGTGCGTGAC-3′, 5′-CAATAGTGATGACCTGGCCGT-3′; Bcl-xl: 5′-GGCATCTTCTCCTYCCAGC-3′, 5′-CCCAGCCTCCGTFATCC-3′; Bcl-2: 5′-GTCGCTACCGTCGTGACTTC-3′, 5′-CAGACATGCACCTACCCAGC-3′; TLR7: ATGTGGACACGGAAGAGACAA, GGTAAGGGTAAGATTGGTGGTG.

### Enzyme-Linked Immunosorbent Assay

Serum levels of IFN-α (ExCell Biology, Shanghai, China) and IFN-β (CUSABIO, Wuhan, China) were detected by ELISA according to the manufacturer's instructions.

### Statistical Analyses

Statistical Package for the Social Sciences (SPSS) software (V.16.0, SPSS Inc., Chicago, IL, USA) was used to analyze the data. All values are presented as the mean ± standard error of the mean (SEM) of three independent experiments. Differences between two groups were assessed using independent-samples *t*-test and differences among more than two groups were conducted using one-way analysis of variance (one-way ANOVA). A *P*-value < 0.05 was considered statistically significant (^*^P < 0.05; ^**^P < 0.01; and ^***^P < 0.001).

## Results

### The Bifunctional Single-Stranded RNA–Pim-3–Small Hairpin RNA Induces Apoptosis of B16F10 Melanoma Cells

**Figure 1:**
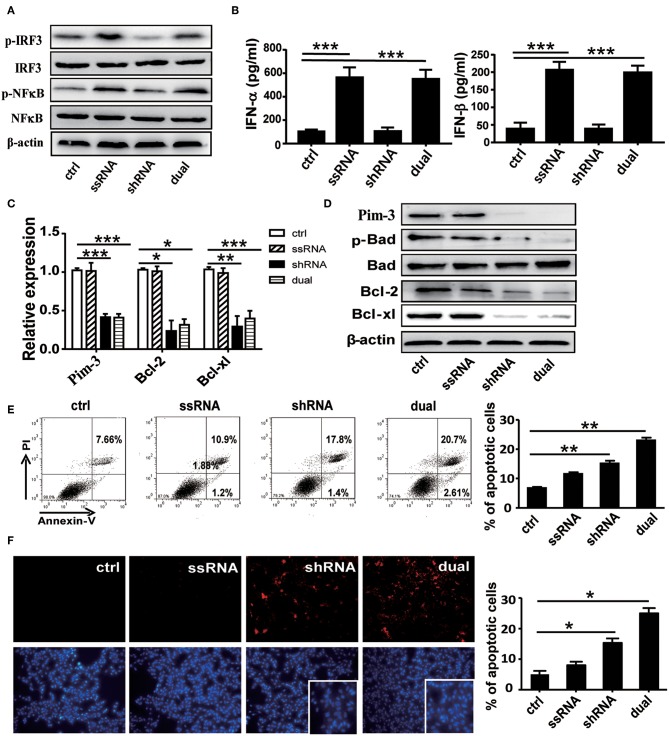
Functional verification of dual-function vector. B16F10 cells transfected with pSIREN (ctrl), ssRNA, sh-Pim-3, or dual-function vector for 24 h. **(A)** Western blot detection of p-IRF3, IRF3, p-NF-κB, NF-κB, and β-actin protein expression. **(B)** Supernatant levels of IFN-α and IFN-β detected by ELISA. **(C)** Gene analysis of Pim-3, Bcl-xl, and Bcl-2 via qRT-PCR in B16F10 cells after transfection for 24 h with the indicated vectors. **(D)** The protein levels of Pim-3, p-Bad, Bcl-xl, and Bcl-2 measured by Western blotting. **(E)** Flow cytometric analysis of apoptosis in B16F10 cells after transfection for 24 h with indicated vectors using annexin V/PI double staining. **(F)** TUNEL staining to evaluate apoptosis of B16F10 cells after transfection for 24 h. Blue fluorescence represents nuclei, and arrowhead indicates shrinking nuclei. Data are representative of three independent experiments. *P < 0.05, **P < 0.01, and ***P < 0.001 vs. control group. ssRNA, single-stranded RNA; IFN, interferon; PI, propidium iodide; TUNEL, terminal deoxynucleotidyl transferase-mediated dUTP nick-end labeling.

We first confirmed the stimulatory effect of ssRNA and dual-function vectors on TLR7 activation. As expected, transfection with the ssRNA and dual-function vectors induced the significant activation of IRF3 and NF-κB that are the downstream signals of TLR7 (Figure 1A) and increased the secreted levels of IFN-α and IFN-β in the supernatants of B16F10 cells (Figure 1B). Further, transfection with sh-Pim-3 and dual-function vectors significantly reduced Pim-3 expression in B16F10 cells at both mRNA and protein levels (Figures 1C,D). Pim-3 is reported to inhibit apoptosis in multiple tumors (4–7). Therefore, we next detected apoptosis of B16F10 cells after transfection with dual-function vector and Pim-3-shRNA vector with annexin V/PI double staining. Silencing of Pim-3 by the dual-function vector and Pim-3-shRNA significantly promoted the apoptosis of B16F10 cells, whereas ssRNA treatment alone had no effect (Figure 1E). We also detected the apoptosis of B16F10 cells via TUNEL staining assay. B16F10 cells, compared with control cells, displayed clearly augmented apoptosis in both Pim-3-shRNA and dual-function vector transfection groups, whereas the apoptosis of B16F10 cells transfected with ssRNA did not change. Shrinking of nuclei and nucleosome production were also observed through nuclear DAPI staining during transfection with the shRNA or dual-function vector. Moreover, the degree of apoptosis was significantly higher in the dual-function vector-transfected group than in the shRNA vector-transfected group (Figure 1F).

Studies have shown that Pim-3 regulates cell apoptosis by inducing the phosphorylation of proapoptotic protein Bad and thus rendering it inactive (6). To understand the mechanisms of Pim-3 regulation of cell apoptosis in B16F10 cells, we detected expression of p-Bad and apoptosis-associated proteins Bcl-xl and Bcl-2 using real-time PCR and Western blotting. We observed that silencing of Pim-3 by the shRNA and dual-function vectors significantly decreased antiapoptotic genes Bcl-xl and Bcl-2 at mRNA and protein levels and significantly suppressed the phosphorylation of Bad but did not affect total levels of Bad (Figures 1C,D). These results suggest that silencing of Pim-3 enhanced B16F10 cells apoptosis by suppressing Bad phosphorylation and reducing Bcl-xl and Bcl-2 expression.

Next, we explored whether Pim-3 silencing affects the proliferation and cell cycle of B16F10 cells. CCK-8 analysis revealed that the proliferation of B16F10 cells was significantly inhibited after transfection with Pim-3-shRNA and dual-function vectors but was not impaired by ssRNA transfection Figure S1A. By flow cytometry–PI staining, we found that transfection of either Pim-3-shRNA or dual-function vector did not affect the cell cycle of B16F10 cells (Figures S1B,C).

Collectively, these results indicate that silencing of Pim-3, particularly by the ssRNA-Pim-3-shRNA dual-function vector, significantly promotes apoptosis and inhibits the proliferation of B16F10 melanoma cells *in vitro*.

### Dual-Functional Vector Therapy Inhibits the Growth of Subcutaneous B16F10 Melanoma *in vivo*

**Figure 2:**
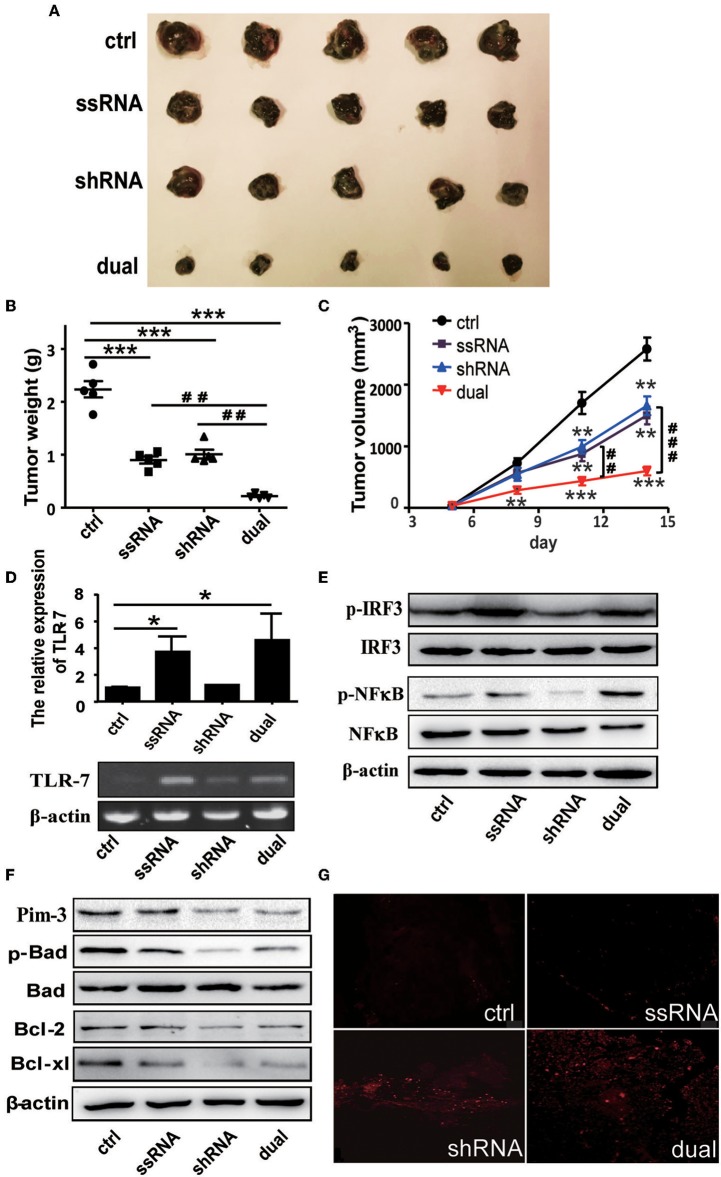
Treatment with bifunctional vector inhibits the growth of B16F10 melanoma *in vivo*. **(A)** Tumor size after subcutaneous challenge of C57BL/6 mice with B16F10 cells and treatment with 10 μg of the indicated vectors by intratumoral injection once every 4 days. **(B)** Statistical analysis of tumor weights after treatment with indicated vectors. **(C)** Statistical analysis of tumor volumes after treatment with indicated vectors. **(D)** Gene analysis of TLR7 via qRT-PCR and PCR in tumor tissue with indicated treatment. **(E)** Western blot detection of p-IRF3, IRF3, p-NF-κB, and NF-κB protein expression of tumor tissue. **(F)** Western blot detection of Pim-3, p-Bad, Bcl-xl, and Bcl-2 protein expression of tumor tissue. **(G)** TUNEL staining to evaluate apoptosis cell of tumor tissue with indicated treatment. Data are representative of three independent experiments. *P < 0.05, **P < 0.01, and ***P < 0.001 vs. control group. ^##^*P* < 0.01 and ^###^*P* < 0.001 vs. dual group. TLR, toll-like receptor; TUNEL, terminal deoxynucleotidyl transferase-mediated dUTP nick-end labeling.

C57BL/6 mice were administered with 5 × 10^5^ B16F10 cells subcutaneously and then were treated with dual-function, shRNA, ssRNA, and control vectors via intratumoral injections every 4 days after the tumor size reached about 50 mm^3^. The tumor volume was measured during 2 weeks of treatment. Tumor growth was significantly inhibited in the ssRNA, shRNA, and dual-function vector treatment groups than in the control group (Figure 2A). As expected, the most significant inhibition effect was observed in the dual-function vector treatment group (Figure 2A). Tumor inhibition is reflected in changes in tumor weight and tumor volume over time by treatment with shRNA, ssRNA, and dual-function vectors; and dual-function vector therapy can better inhibit tumor growth than do ssRNA and shRNA (Figures 2B,C). No significant difference was observed between the ssRNA and shRNA treatment groups (Figures 2A–C).

Next, we examined the changes of TLR7 activation and Pim-3-silencing-related apoptosis of tumor tissues after different treatment. As expected, ssRNA and dual-function vector therapy significantly promoted the expression of TLR7 and the phosphorylation of IRF3 and NF-κB in tumor tissues (Figures 2D,E). Further, Western blotting results showed that the expression of Pim-3 in tumor tissues was significantly reduced after shRNA and dual-function vector therapy. Also, the phosphorylation of Bad and apoptosis-associated proteins Bcl-xl and Bcl-2 was markedly decreased in shRNA and dual-function vector therapy groups (Figure 2F). TUNEL staining assay showed that shRNA and dual-function vector therapy significantly promoted the apoptosis of tumor cells, whereas ssRNA treatment alone had no effect (Figure 2G).

These results indicate that whether by silencing of Pim-3 by shRNA or stimulation, the activation of TLR7 by ssRNA can inhibit the growth of melanoma. Importantly, treatment with dual-functional vector, which has the capacity to both silence Pim-3 and stimulate the TLR7 pathway, can achieve more significant antitumor efficacy.

### Treatment With Dual-Function Vector Enhances the Activation of Natural Killer Cells and CD8^+^ T Cells *in vivo*

**Figure 3:**
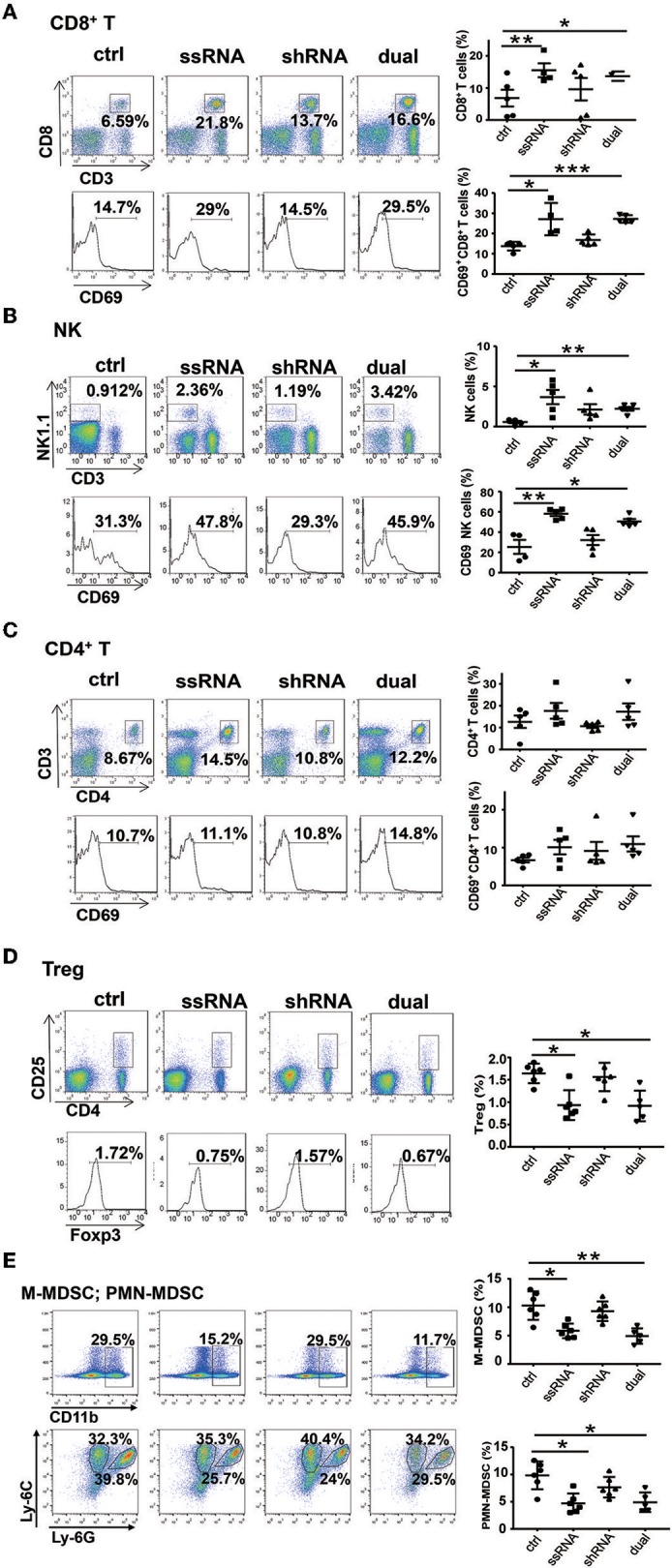
Treatment with bifunctional vector enhances the activation of NK and CD8^+^ T cells *in vivo*. The percentages and activation of tumor-infiltrating leukocytes in C57BL/6 mice was determined via flow cytometric analysis. **(A)** CD8^+^ T cells. **(B)** NK cells. **(C)** CD4^+^ T cells. **(D)** Treg cells. **(E)** M-MDSCs and PMN-MDSCs. Data are representative of three independent experiments. *P < 0.05, **P < 0.01, and ***P < 0.001 vs. control group. NK, natural killer; Treg, regulatory T cell; M-MDSC, monocytic myeloid-derived suppressor cell; PMN, polymorphonuclear.

As described above, both ssRNA and Pim-3 shRNA inhibit the growth of melanoma. Because ssRNA activates the TLR7 signaling pathway, we evaluated whether ssRNA treatment further stimulates immune responses. We examined the percentages and activation of different immune cells in tumor tissues and spleen of tumor-bearing mice by flow cytometry; the gating strategy of different cells is shown in Figure S2. The results showed that the percentages and activation of CD8^+^ T cells and NK cells in the melanoma tissues were significantly increased after treatment with ssRNA and dual-function vectors (Figures 3A,B), whereas the proportions and activation of CD4^+^ T cells did not change (Figure 3C). Interestingly, the proportions of immunosuppressive Treg cells, monocytic MDSCs (M-MDSCs), and polymorphonuclear (PMN)-MDSCs in tumor tissues were significantly reduced via treatment with ssRNA and dual-function vectors (Figures 3D,E). Treatment with shRNA alone had little effect on these intratumor immune cells compared with those in the control group (Figures 3A–E). Similarly, ssRNA and dual-function vectors markedly augmented the proportion and activation of CD8^+^ T cells and NK cells in the spleen, whereas CD4^+^ T cells showed no significant changes (Figures S3A–C). However, the percentages of Treg cells were significantly reduced via treatment with ssRNA and dual-function vectors in the spleen (Figure S3D). These results demonstrate that ssRNA and dual-function vectors enhance the proliferation and activation of NK and CD8^+^ T cells in both systemic and local tumor tissues and reduce the proportions of immunosuppressive cells, thus not only priming activation of antitumor immune responses but also reversing the immunosuppressive tumor microenvironment. The antitumor efficacy of shRNA may be mainly dependent on the apoptosis of tumor cells caused by silencing of Pim-3.

### Natural Killer and CD8^+^ T Cells Are Required for the Dual-Function Vector-Mediated Antitumor Effect

**Figure 4:**
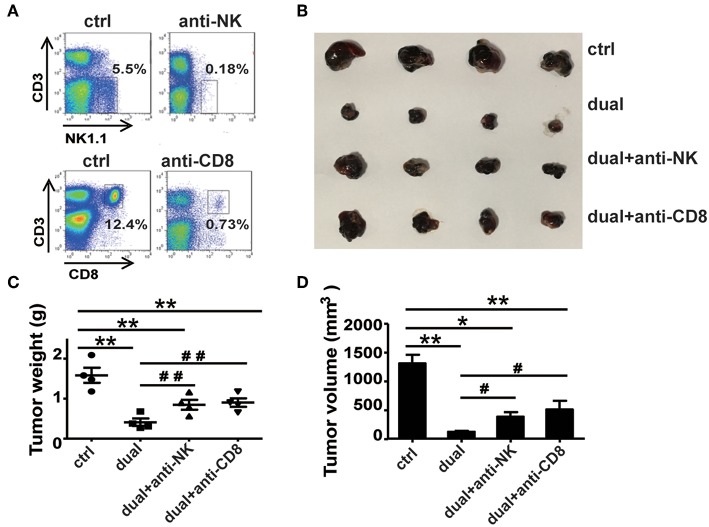
NK and CD8^+^ T cells are necessary for the treatment effect of the dual-function vector on B16F10 melanoma. **(A)** The effect of PK136 and 2.43 antibodies on NK and CD8^+^ T cell depletion. **(B)** Tumor sizes after treatment. **(C)** Analysis of tumor weights. **(D)** Analysis of tumor volumes. Data are representative of three independent experiments. **P < 0.01 or *P < 0.05 vs. control group. ^#^*P* < 0.05 and ^##^*P* < 0.01 vs. dual group. NK, natural killer.

From the above results, we observed a significant increase in the proportions and activity of NK cells and CD8^+^ T cells, whereas CD4^+^ T cells did not affect the ssRNA and dual-function vector-treated mice. These findings suggest that CD8^+^ T cells and NK cells might be the main effector cells responsible for the ssRNA-induced antitumor immune responses. To test this hypothesis, we depleted NK cells, CD8^+^ T cells, and CD4^+^ T cells by administering mAb PK136 (anti-NK1.1), 2.43 (anti-CD8a), and GK1.5 (anti-CD4) intraperitoneally every 3 days, respectively, before the mice were inoculated with tumor cells. The dual-function vector was then administered, and melanoma growth was monitored. Figure 4A and Figure S4A show that the depleting antibodies almost completely depleted NK cells, CD8^+^ T, and CD4^+^ T cells in mice. As expected, dual-function vector treatment significantly inhibited the growth of tumors (Figures 4B–D). It is notable that both NK and CD8^+^ T cell depletion significantly impaired the dual-function vector-induced tumor suppression (Figures 4B–D), whereas administering giving anti-CD4 antibody did not affect dual-function vector therapy (Figures S4B–D). These results suggest that both NK cells and CD8^+^ T cells but not CD4^+^ T cells are required for the dual-function vector-mediated antitumor immune responses against melanoma.

### Plasmacytoid Dendritic Cells Are Involved in the Activation of Natural Killer and CD8^+^ T Cells

**Figure 5:**
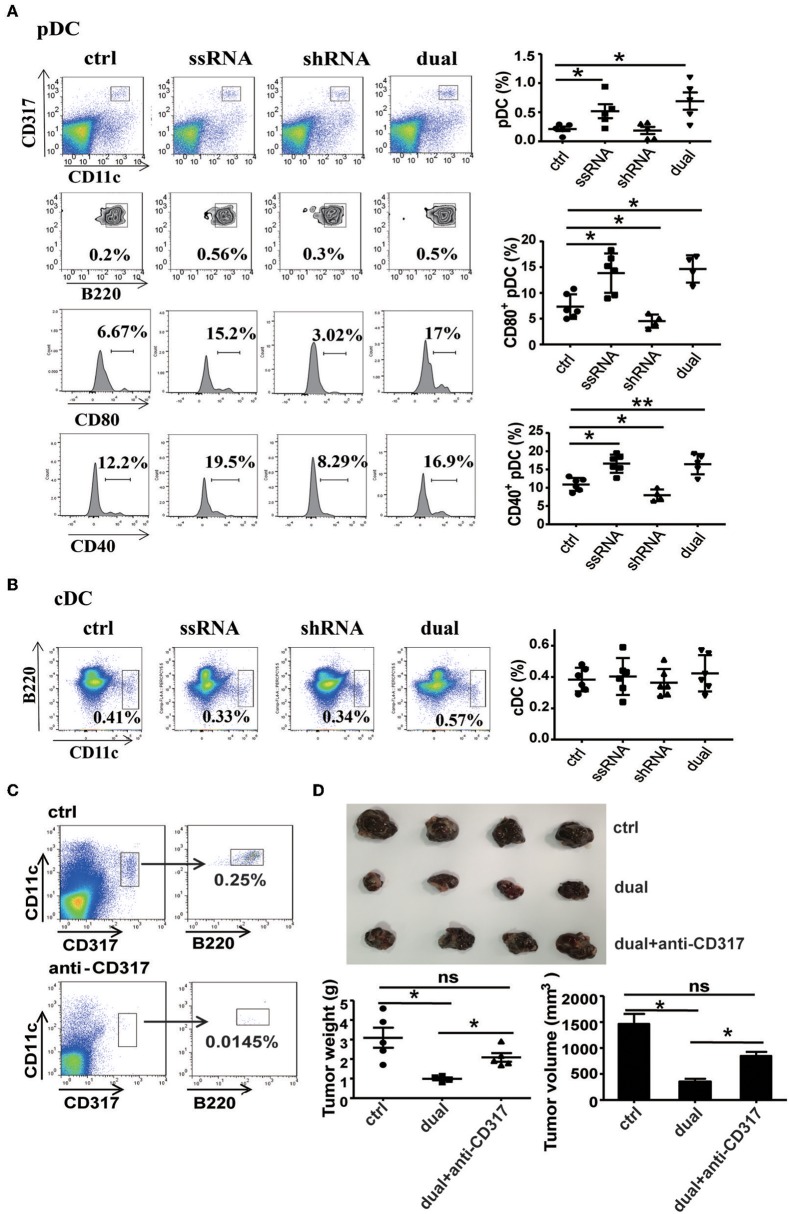
pDCs are necessary for the treatment effect of the dual-function vector on B16F10 melanoma. **(A)** The percentages and activation of pDCs in tumor tissues of C57BL/6 mice were determined via flow cytometric analysis. **(B)** The percentages of cDCs in tumor tissues of C57BL/6 mice were determined via flow cytometric analysis. **(C)** pDC depletion effect of anti-CD317 antibody. **(D)** Image shows tumor sizes. And the tumor weight and volume were analyzed. Data are representative of three independent experiments. **P < 0.01 or *P < 0.05 vs. control group. pDCs, plasmacytoid dendritic cells.

ssRNA specifically binds to TLR7 and activates downstream signaling pathways to induce type I IFN production (11–13). As a major producer of type I IFN, pDCs exert an effective response to tumors and viral infection mainly by secreting type I IFN (18). It has been reported that TLR7 agonist 852A can stimulate pDCs to secrete abundant type I IFN and thus inhibit the proliferation of tumor cells (21). To determine the role of pDCs in ssRNA- and dual-function vector-mediated antitumor immune responses, we examined the proportion and activation of pDCs in spleen and tumor tissues of melanoma-bearing mice. Flow cytometric analysis revealed that ssRNA and dual-function vector treatment significantly increased the proportion of pDCs (CD317^+^CD11c^+^B220^+^) in tumor tissues (Figure 5A) and also enhanced the expression of CD80 and CD40 on pDCs, which suggest the activation of pDCs. Similarly, ssRNA and dual-function vectors markedly augmented the proportion and activation of pDCs in the spleen (Figure S5A), whereas cDCs showed no significant changes in both tumor sites and spleen (Figure 5B and Figure S5B). Notably, the expression levels of CD80 and CD40 on pDCs in tumor sites decreased in the shRNA-treated group (Figure 5A), whereas the activation of pDCs in spleen was not impaired (Figure S5A), suggesting that shRNA-induced tumor apoptosis might suppress the activation of pDCs. To further verify the role of pDCs in dual-function vector therapy, we depleted pDCs with an anti-CD317 antibody and then observed the effect of dual-function vector therapy on melanoma growth. Figure 5C shows that anti-CD317 antibody treatment can effectively deplete pDCs (Figure 5C). Notably, the therapeutic effect of the dual-function vector was significantly attenuated by pDC depletion (Figure 5D). These results clearly demonstrate that pDCs are essential for the dual-function vector-mediated antitumor effect.

**Figure 6:**
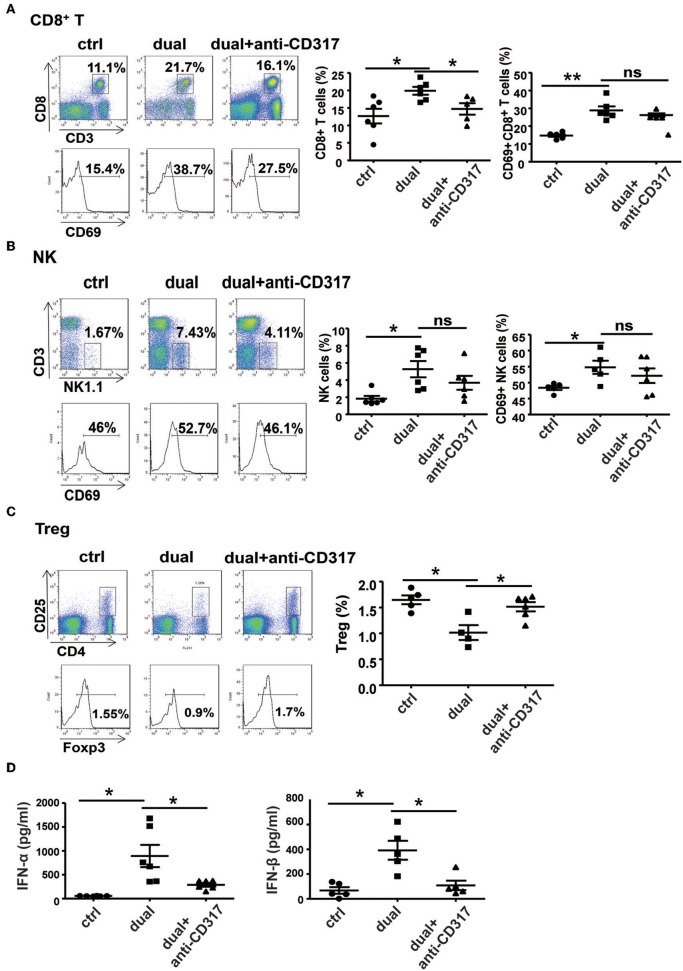
pDCs are necessary for the activation of NK cells and CD8^+^ T cells *in vivo*. The percentages and activation of CD8^+^ T cells **(A)**, NK cells **(B)**, and Treg cells **(C)** in tumor tissues after pDC depletion were determined by flow cytometric analysis. **(D)** Serum levels of IFN-α and IFN-β were detected by ELISA after pDC depletion. Data are representative of three independent experiments. **P < 0.01 or *P < 0.05 vs. control group. pDCs, plasmacytoid dendritic cells; NK, natural killer; Treg, regulatory T cell; IFN, interferon.

To observe the relationship between pDCs and NK cells and CD8^+^ T cells during dual-function vector-mediated antitumor immune responses, we analyzed the proportion and activation state of NK cells and CD8^+^ T cells in tumor tissues and spleen after treatment with dual-function vector and elimination of pDCs. The dual-function vector significantly increased the percentages and activation of CD8^+^ T cells and NK cells in tumor tissues as described above (Figures 6A,B). However, after depletion of pDCs, the percentage of CD8^+^ T cells in tumor sites was decreased compared with that in the dual-function vector-treated group (Figure 6A). Furthermore, the percentages and activation of both NK cells and CD8^+^ T cells in the spleen stimulated by dual-function vector were significantly reduced after pDCs were depleted (Figures S6A,B). Moreover, we found that depletion of pDCs restored Treg cell percentages in both tumor tissues and spleen in the dual-function vector-treated group (Figure 6C and Figure S6C). Further, we examined whether the secretion of type I IFN was affected after pDC depletion. As expected, therapy with dual-function vector significantly augmented the serum levels of IFN-α and IFN-β, which markedly reduced in the pDC depletion group (Figure 6D). These results suggest that dual-function vector may first stimulate pDCs to produce type I IFNs, which in turn activate NK cells and CD8^+^ T cells accompanied by suppression of Treg and MDSCs, thus priming NK- and CD8^+^ T cell-mediated antitumor immune responses and inhibiting tumor growth in combination with Pim-3 shRNA-mediated tumor cell apoptosis.

## Discussion

**Figure 7:**
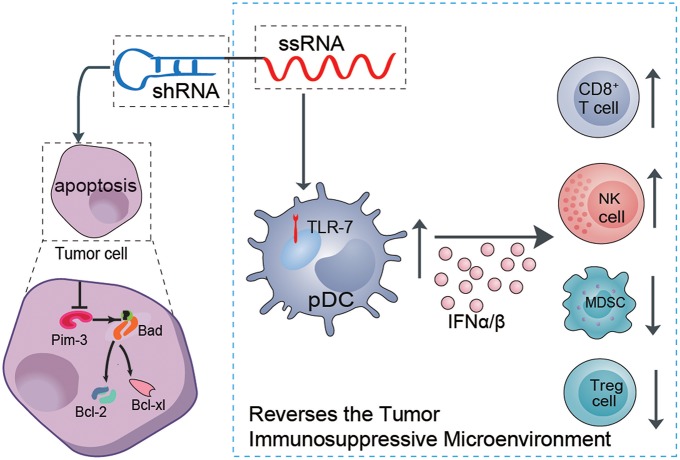
Schematic illustration of the role of the dual ssRNA-Pim-3-shRNA vector with both Pim-3-silencing and immunostimulatory effects for inhibiting melanoma growth. Therapy with the dual-function vector not only promotes the apoptosis of B16F10 melanoma cells by silencing Pim-3 but also activates pDCs by ssRNA, leading to secretion of a large amount of type I interferon, enhanced activation of NK cells and CD8^+^ T cells, and reduced percentages of Tregs and MDSCs. Combined, these effects reverse the tumor immunosuppressive microenvironment and inhibit the growth of melanoma cells. ssRNA, single-stranded RNA; shRNA, small hairpin RNA; pDCs, plasmacytoid dendritic cells; NK, natural killer; Treg, regulatory T cell; MDSCs, myeloid-derived suppressor cells.

Some proto-oncogenes boost proliferation and inhibit apoptosis of melanoma cells, which are the main etiologies of tumor development. Although melanoma is considered a prototypical immunogenic tumor, efficient antitumor immune responses usually cannot be mounted in melanoma patients. There is usually an insufficient innate immune response to initiate specific adaptive antitumor immunity. In addition, the dominant immunosuppressive microenvironment (i.e., immunosuppressive cytokines and suppressor cells) induced by the tumor itself leads to immune cell dysfunction or exhaustion, with failure to control and eliminate melanoma cells (22–24). Thus, effective anti-melanoma therapy must both suppress oncogene expression to inhibit proliferation or promote the apoptosis of melanoma and simultaneously restimulate or reawaken suppressed anti-immune responses. Using our previously established ssRNA-sh-Pim-3 dual-function vector, which both silences the proto-oncogene Pim-3 to promote apoptosis of tumor cells and simultaneously activates TLR7 to stimulate antitumor immune responses (8, 25), we verified that the dual-function vector not only promotes apoptosis and inhibits proliferation of B16F10 melanoma cells but also stimulates the activation of pDCs to secrete a large amount of type I IFN. This leads to enhanced activation of CD8^+^ T cells and NK cells and reduces the percentages of Tregs and MDSCs and thus effectively reverses the tumor immunosuppressive microenvironment and inhibits melanoma growth (Figure 7).

Although T cells can infiltrate into melanoma tumor sites directed by chemokines, they subsequently become functionally inhibited by the immunosuppressive microenvironment, including the high expression of programmed cell death ligand 1 (PD-L1) on tumor cells, the high infiltration with Treg cells or MDSCs, and the highly secreted factors TGF-β, IL-10, and IDO. These factors hinder the function of intratumoral T cells, rendering them dysfunctional or anergic and thus ultimately allow tumor escape and outgrowth (26–29). Our present study used an ssRNA-sh-Pim-3 dual-function vector to treat melanoma and showed that this therapeutic strategy can subvert tumor-induced immunosuppression by inducing activation of both NK and CD8^+^ T cells and reducing the percentages of Tregs and MDSCs in the tumor microenvironment. Intriguingly, this therapy not only reversed the local antitumor immune responses in tumor sites but also restored systemic antitumor immunity as shown by the increased frequency and activation of splenic CD8^+^ T cells and NK cells accompanied with reduced Treg cells (Figure S3). Our former research has shown a similar antitumor effect of this dual-function vector for treatment of subcutaneous Hepa1-6 cell hepatoma, which also has shown that the dual-function vector inhibited the growth of hepatoma more efficiently than did single shRNA or ssRNA treatment *in vivo* (8). However, the immunological mechanism seems different. In the hepatoma model, treatment with the dual-function vector significantly promoted the activation of NK and CD4^+^ T cells. NK and CD4^+^ T cells, but not CD8^+^ T cells, were essential to dual-function therapy-mediated effective tumor suppression, and CD4^+^ T cells provided help for the activation of NK cell by production of Th1 or Th2 cytokines. In contrast, the present study showed that, in a melanoma model, the main effect of dual-function vector is to stimulate the activation of pDCs to secrete a large amount of type I IFN, which further enhances the activation and antitumor effect of CD8^+^ T and NK cells. CD4^+^ T cells seem to not play a major role in this model. The result suggests that there are distinct therapeutic mechanisms for the dual-function vector in different tumor microenvironment.

The activation and function of innate immune system and DC subsets play a critical role in priming and bridging toward antitumor adaptive immune responses (30, 31). Type I IFN signaling participates in the innate recognition of tumors and also contributes to the priming of CD8^+^ T and NK cells (11, 13). However, several lines of evidence have shown that accumulation of some DC subsets is nearly absent or become tolerogenic in the tumor microenvironment including melanoma (17, 26). pDCs usually exert antiviral activity and facilitate protective immune response of both innate and adaptive immunity through the secretion of large amounts of type I IFN upon TLR stimulation (32, 33). Through production of IFN-α, pDCs promote the activation and migration of NK cells;, he stimulation of macrophage and dendritic cells; and the activation, survival, and expansion of CD8^+^ T cells (33, 34). They also promote the adaptive immune response of both CD4^+^ and CD8^+^ T cells by acting as APCs (35, 36). Several reports have shown that pDCs have the potential to induce antitumor immunity and can be harnessed to elicit antigen-specific antitumor immune responses *in vivo* (37, 38). In the present study, we found that therapy with ssRNA-sh-Pim-3 dual-function vector initiates the activation of pDCs through ssRNA-mediated stimulation of the TLR7 signaling pathway. Activated pDCs secrete plenty of type I IFN and thus effectively prime the activation of CD8^+^ T and NK cells to exert antitumor efficacy. Importantly, the therapeutic effect of the bifunctional vector was significantly attenuated after pDC depletion (Figure 5). Also, the degree of NK and CD8^+^ T cell activation and the production of type I IFN were significantly reduced, whereas the percentages of Treg cells in tumor sites were increased after pDC depletion (Figure 6). These results clearly demonstrate that bifunctional therapy can reverse the tumor immunosuppressive microenvironment in a pDC-dependent manner. Other reports have also explored the exploitation of pDCs to induce antitumor immunity. For example, TLR9 agonist CpG oligodeoxynucleotide (ODN) multimers can induce pDCs to produce IFN-α and further enhance the ability of NK and CD8^+^ T cells to eradicate the established tumors (39, 40).

However, there is also emerging evidence demonstrating that tumor-infiltrating pDCs tend to be tolerogenic rather than immunogenic and are often associated with poor clinical outcomes in several types of cancer, including melanoma (33, 35). Tumor-infiltrating pDCs display an impaired response to TLR7/9 activation and decreased or defective production of type I IFN, and contribute to the establishment of an immunosuppressive tumor microenvironment through high expression of PD-L1 and IDO (36, 41). It has been previously shown that pDCs are affected by an apoptotic cell-enriched microenvironment (42). In the present study, we observed that treatment with either shRNA vector or dual-function vector induced significant apoptosis of melanoma cells in tumor tissues (Figure 2G). Through analyzing the activation of intratumoral pDCs, we found that shRNA treatment decreased the activation of pDCs (with decreased expression levels of CD80 and CD40 on pDCs in tumor sites), whereas therapy with both ssRNA and dual-function vector, compared with the control group, significantly enhanced the activation of intratumoral pDCs (Figure 5A). These results indicated that shRNA-induced tumor apoptosis might impair the activation of pDCs, whereas adding ssRNA might counteract the potential deleterious immunosuppressive effect of increased apoptotic melanoma cells. Moreover, reactivation of pDCs by ssRNA and dual-function vector therapy also reverses the tumor immunosuppressive microenvironment by promoting the activation of NK cells and CD8^+^ T cells and reduces the percentages of Tregs and MDSCs in tumor sites. We propose that this effect may result from the production of type I IFN, although the exact mechanism needs further investigation. Recent studies reported that TLR8 signaling reversed Treg suppression by selectively inhibiting glucose uptake and glycolysis in human Treg cells, and it enhanced antitumor immunity (14, 43, 44). These findings further demonstrate the significant potential of therapeutic activation of tumor pDCs through stimulating TLR signaling pathway in tumor immunotherapy.

In summary, we have shown that therapy with ssRNA-Pim-3-shRNA dual-functional vector not only induces the apoptosis of B16F10 melanoma cells by silencing Pim-3 but also stimulates pDCs to secrete a large amount of type I IFN via ssRNA activation and thus enhances activation of NK cells and CD8^+^ T cells. Together, these activities effectively inhibit the growth of melanoma. Importantly, this therapy can reverse the tumor immunosuppressive microenvironment by counteracting apoptosis-mediated inactivation of pDCs, reducing the percentages of Tregs and MDSCs, and facilitating the activation of both NK and CD8^+^ T cells. Our study suggests that this dual-function therapeutic strategy might become a promising modality in the future therapy for melanoma or other related solid tumors.

## Data Availability Statement

The raw data supporting the conclusions of this manuscript will be made available by the authors, without undue reservation, to any qualified researcher.

## Ethics Statement

This animal study was reviewed and approved by Shandong University Committee on the Ethics of Animal Experiments.

## Author Contributions

JL designed and performed experiments, analyzed data, and wrote the manuscript. YH, XY, QG, and LS performed experiments and analyzed data. CZ conceived and supervised the study, designed experiments, analyzed and interpreted data, and wrote the manuscript. All authors read and approved the final manuscript.

### Conflict of Interest

The authors declare that the research was conducted in the absence of any commercial or financial relationships that could be construed as a potential conflict of interest.
