# Supplementary material for: Enhanced Anti-melanoma Efficacy of a Pim-3-Targeting Bifunctional Small Hairpin RNA via Single-Stranded RNA-Mediated Activation of Plasmacytoid Dendritic Cells
Source: Front Immunol. 2019 Nov 26;10:2721. doi: 10.3389/fimmu.2019.02721 (PMC6902031; doi:10.3389/fimmu.2019.02721)
Supplement: Supplementary file 1 [file Data_Sheet_1.pdf]

Enhanced anti-melanoma efficacy of a Pim-3-targeting bifunctional shRNA via ssRNA-mediated activation of pDCs

Jing Liu, Yuan Hu, Qie Guo, Xin Yu, Liwei Shao, Cai Zhang*

Institute of Immunopharmaceutical Sciences, School of Pharmaceutical Sciences, Shandong University, Jinan, Shandong 250012, China

Supplementary Table 1. Antibodies used for flow cytometry

Fluorophore	Antibody	Clone name	Company
APC	anti-mouse CD69	H1.2F3	eBioscience
Percp-cy5.5	anti-mouse CD8α	H35-17.2	eBioscience
PE	anti-mouse CD4	RM4-5	eBioscience
Percp-cy5.5	anti-mouse CD4	RM4-5	eBioscience
PE-Cy7	anti-mouse CD3e	145-2C11	eBioscience
APC-cy7	anti-mouse NK1.1	PK136	eBioscience
FITC	anti-mouse CD317	927	BioLegend
Percp-cy5.5	anti-mouse B220	RA3-6B2	eBioscience
BV510	anti-mouse CD11c	N418	BioLegend
PE	anti-mouse CD25	PC61	eBioscience
APC	anti-mouse Foxp3	FJK-16s	eBioscience
FITC	anti-mouse CD11b	ICRF44	BioLegend
PE	anti-mouse ly6G	1A8	BioLegend
APC	anti-mouse ly6C	HK1.4	BioLegend
PE	anti-mouse CD80	16-10A1	BioLegend
APC	anti-mouse CD40	3/23	BioLegend
PE-CF594	anti-mouse CD45	30-F11	BioLegend
BV421	Fixable Viability Dye		BioLegend
BV510	Fixable Viability Dye		BioLegend

Supplementary Figures

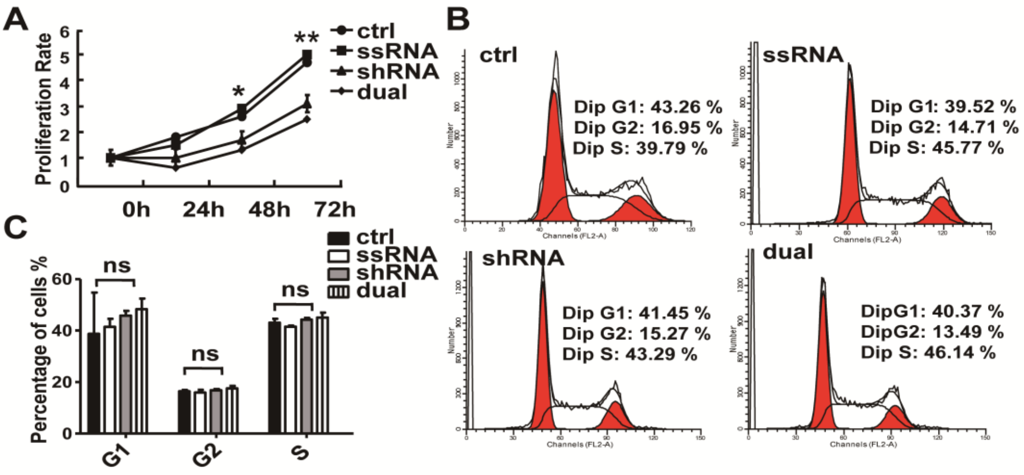

Figure S1. The proliferation and cell cycle status of B16F10 cells were detected after transfection with different vectors. (A) Cell proliferation of B16F10 cells after transfection for 24 hours with the indicated vectors. **(B)** Flow cytometric analysis of B16F10 cell cycle stage after transfection for 24 hours. **(C)** Statistical analysis of B16F10 cell cycle stages. Data are representative of three independent experiments. **P < 0.01 or *P < 0.05 versus control group.

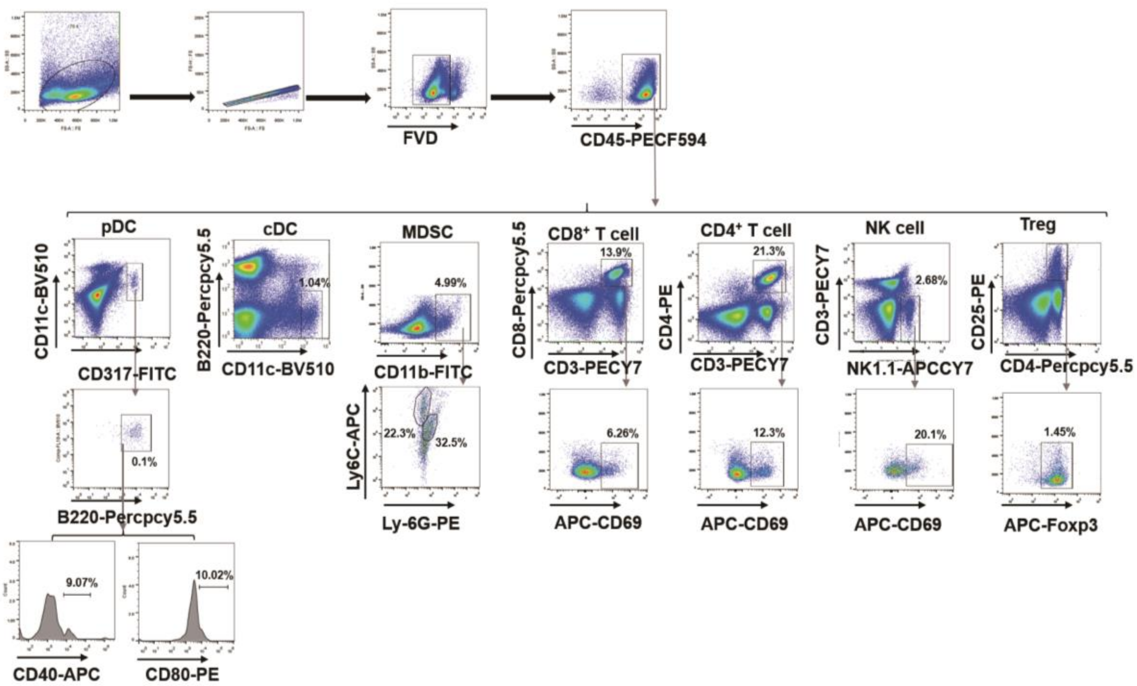

Figure S2. FACS gating strategy. FSA x SSA gating to obtain cells based on size and granularity. Single cells were identified by plotting forward scatter-area against forward scatter-height and were then separated from debris by a forward versus side scatter. Then live/dead FVD marker was used to identify live cells. Leucocytes were gated on CD45-postive events. Staining with lineage-specific antibodies against $CD4^{+}$T cells ($CD3^{+}CD4^{+}$, activation markers: CD69), $CD8^{+}$T cells ($CD3^{+}CD8^{+}$, activation markers: CD69), NK cells ($NK1.1^{+}CD3^{-}$, activation markers: CD69), pDCs ($CD317^{+}CD11c^{int}B220^{hi}$, activation markers: CD80, CD40), cDCs ($CD11c^{hi}B220^{-}$), MDSCs (M-MDSCs: $CD11b^{+}Ly6C^{+}Ly6G^{neg}$, PMN-MDSCs: $CD11b^{+}Ly6C^{neg}Ly6G^{+}$) and Treg cells ($CD4^{+}CD25^{+}Foxp3^{+}$) to allow identify of these cells and detect its function. The proportion of pDCs and Treg displayed is calculated as a percentage of CD45 positive cells.

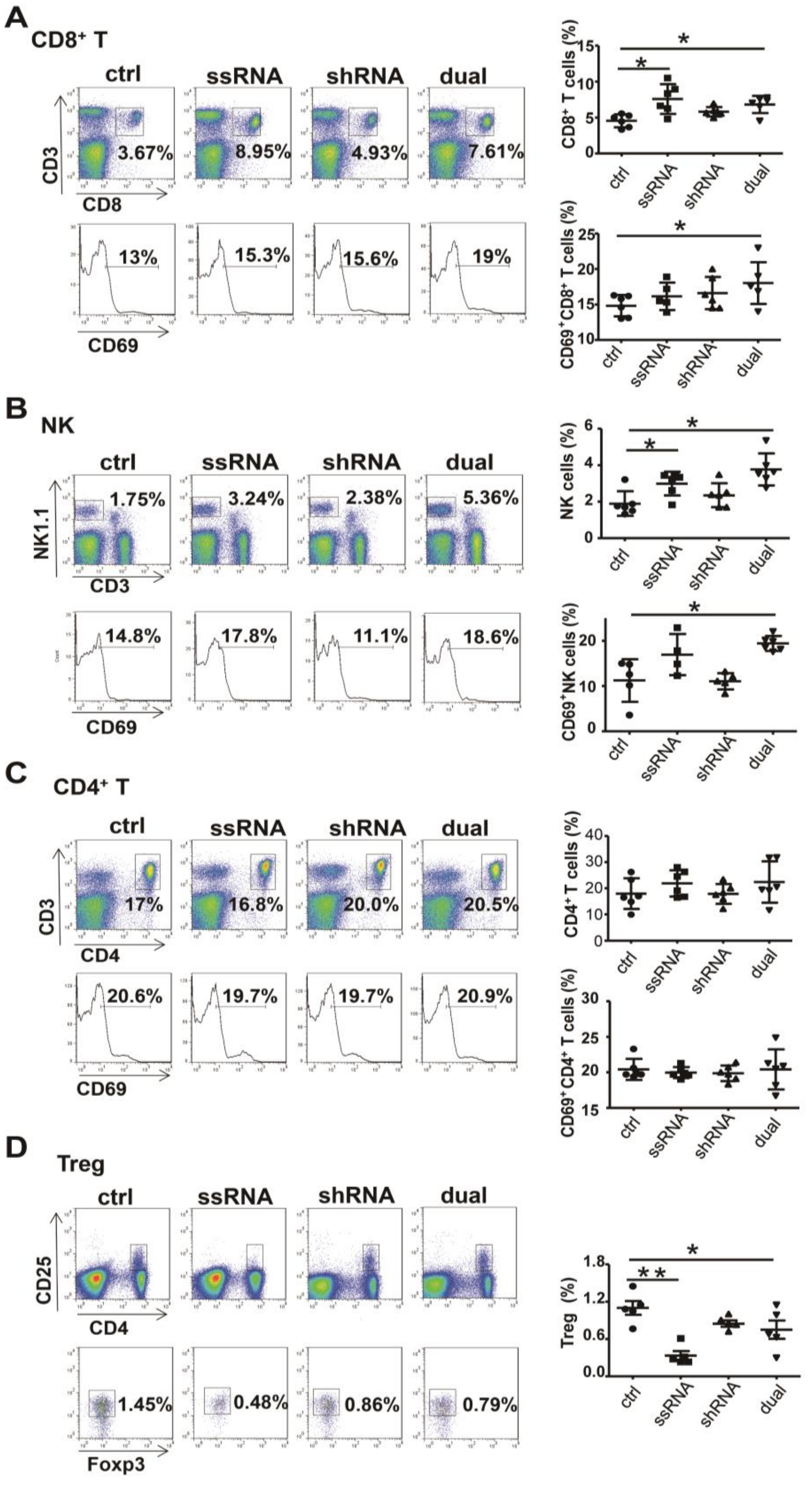

Figure S3. Treatment with bi-functional vector enhances the activation of NK and CD8+ T cells in spleen. The percentages and activation of spleen in

tumor-bearing C57BL/6 mice were determined via flow cytometric analysis. **(A)** $CD8^+$ T cells. **(B)** NK cells. **(C)** $CD4^+$ T cells. **(D)** Treg cells. Data are representative of three independent experiments. **P* < 0.05, ***P* < 0.01, ****P* < 0.001 versus control group.

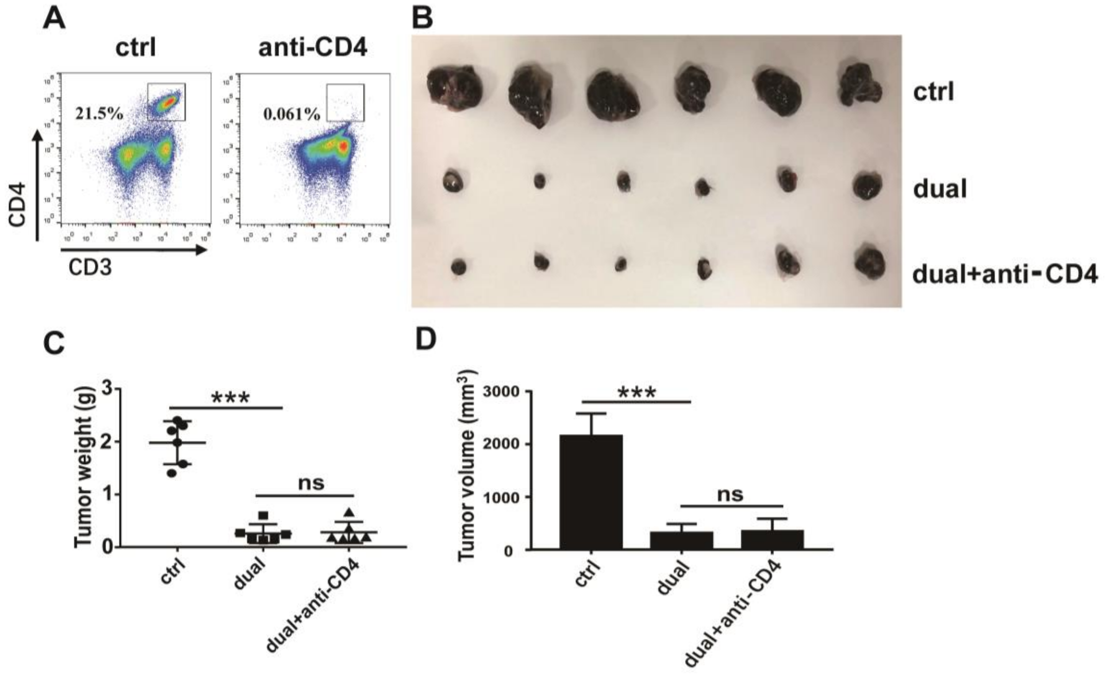

Figure S4. Depletion of $CD4^+$ T cells for the treatment effect of the dual-function vector on B16F10 melanoma. (A) The effect of $CD4^+$ T cell depletion by injection of GK1.5 antibody. **(B)** Tumor sizes after treatment. **(C)** Analysis of tumor weights. **(D)** Analysis of tumor volumes. Data are representative of three independent experiments. ****P* < 0.001 versus control group.

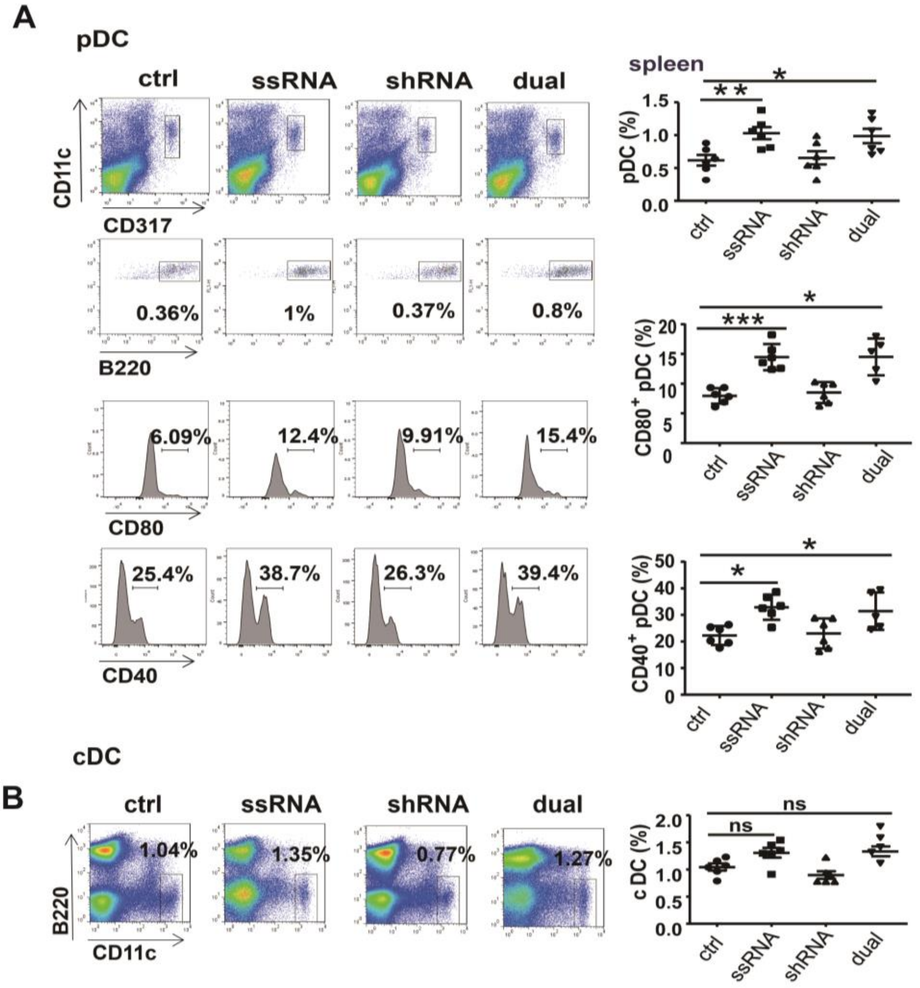

Figure S5. The percentages and activation of pDCs were increased in spleen. (A) The percentages and activation of pDCs in spleen of C57BL/6 mice were determined via flow cytometric analysis. **(B)** The percentages of cDCs in spleen of C57BL/6 mice were determined via flow cytometric analysis. Data are representative of three independent experiments. **P* < 0.05, ***P* < 0.01, ****P* < 0.001 versus control group.

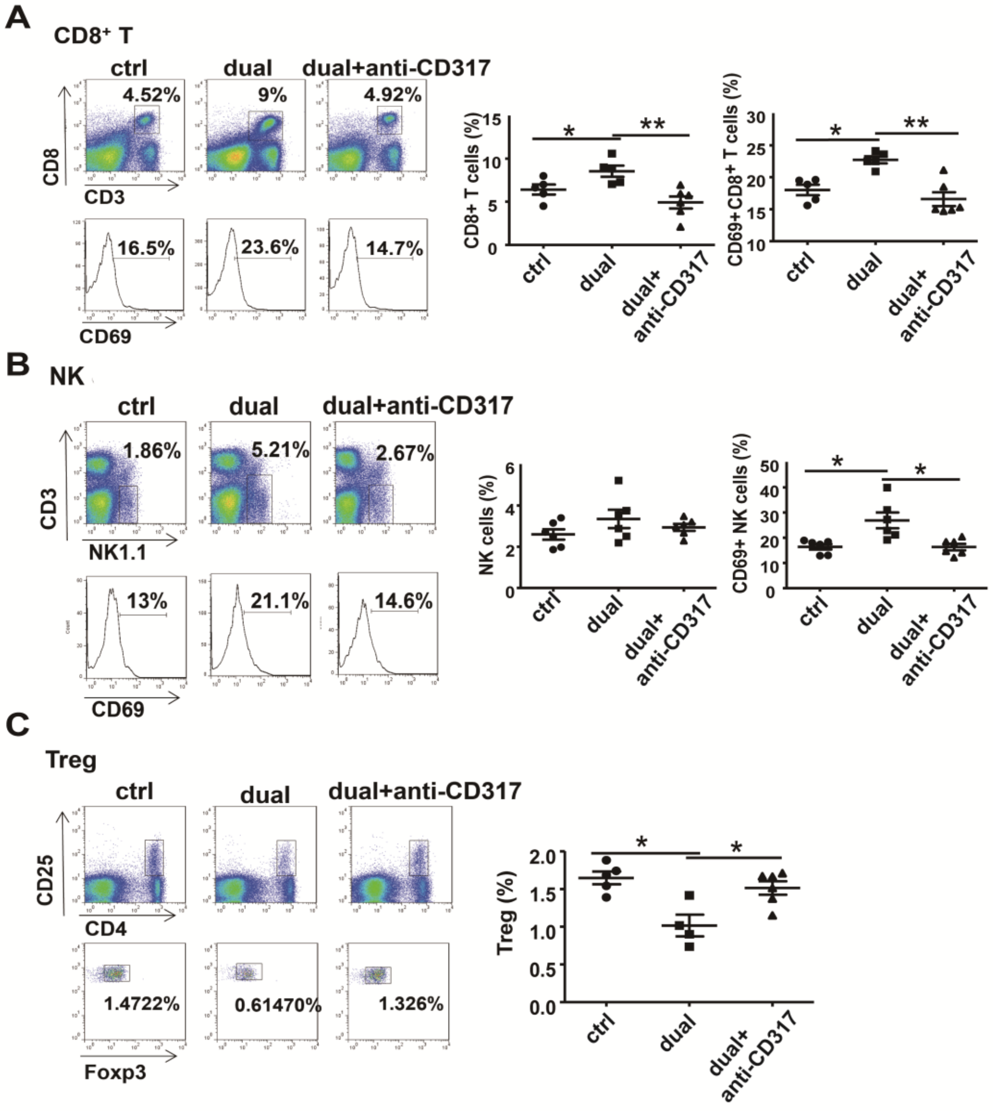

Figure S6. pDCs are necessary for the activation of NK cells and CD8$^+$ T cells in spleen. The percentages and activation of CD8$^+$ T cells **(A)**, NK cells **(B)** and Treg cells **(C)** in spleen after pDC depletion were determined by flow cytometric analysis. Data are representative of three independent experiments. ***P* < 0.01 or **P* < 0.05 versus control group.
